# Blood lipidome profiling reveals potential biomarkers linked to health and carcass quality traits in pigs

**DOI:** 10.1186/s12711-026-01030-3

**Published:** 2026-01-30

**Authors:** Carles Hernández-Banqué, Teodor Jové-Juncà, Manel Portero-Otin, Elia Obis, Olga González-Rodríguez, Maria Ballester, Raquel Quintanilla

**Affiliations:** 1 Animal Breeding and Genetics Program, IRTA, Torre Marimon, 08140 Caldes de Montbui, Barcelona, Spain; 2 Department of Experimental Medicine, University of Lleida-Biomedical Research Institute of Lleida, 25198 Lleida, Spain

## Abstract

**Background:**

The modulation, activation, and differentiation of several immune cells is highly dependent on lipid metabolism. The objective of this study was to analyse the genetic determinism of the porcine plasma lipidome and its association to the animal immune capacity and production performance. The analysis of the blood lipidome of 300 60-day-old Duroc pigs allowed semi-quantification of 982 circulating lipid molecules. We evaluated the genetic determinism of the lipidome abundances, as well as their phenotypic and genetic correlations with health, stress, and carcass phenotypes.

**Results:**

Triacylglycerols were the most abundant lipid class among the plasma lipid features, followed by glycerophosphocholines, glycerophosphoethanolamines, diacylglycerols, and fatty acids/esters. Lipidome abundances showed low to moderate phenotypic correlations with the health and production traits, which clustered in two groups with opposite phenotypic correlation patterns with the lipidome. Mean heritability estimates for the circulating lipids abundance was generally low, but 184 lipid molecules showed significant heritability ranging between 0.25 and 0.85. At the genetic level, the percentage and phagocytic capacity of lymphocytes, the proportion of γδ T lymphocytes, and the cortisol concentration in hair were especially correlated with the lipidome, showing more than 200 significant genetic correlations with different lipidic compounds. Putative identification of associated metabolites by mass similarity revealed a large presence of phospholipids and glycerolipids among lipid molecules genetically correlated with immunity traits. Regarding production traits, fatness and lean meat measures showed an opposite pattern of genetic correlations with the porcine lipidome. Lipids positively correlated with fatness were mainly composed of diacyl- and triacyl-glycerides, while potential ceramides and phospholipids were more abundant among the lipids positively correlated with lean meat content at the genetic level.

**Conclusions:**

Our results demonstrate a genetic determinism of the porcine blood lipidomic profile and suggest genetic correlations of the lipidome abundances with health and production performance phenotypes. We identify potential lipid biomarkers for assessing animal health and productivity.

**Supplementary Information:**

The online version contains supplementary material available at 10.1186/s12711-026-01030-3.

## Background

The porcine industry is facing the need to reduce the use of antibiotics while maintaining farm productivity and health [1, 2]. Besides the already established measures such as vaccination and biosecurity protocols, improving the immunocompetence of pig populations through genetic selection has shown to be a promising approach [3–5]. Global immunocompetence of healthy animals can be measured through the determination of immunity traits, encompassing innate and adaptive immunity. Both innate and adaptive immunity mechanisms are regulated by several metabolic processes, while immune cells per se have the capacity to modulate the function of metabolic organs by shifting metabolic pathways and requirements. Recent research in the field of immunometabolism has highlighted the importance of small molecules and metabolic intermediates during immune activation, showing the potential to affect the immune response by targeting specific immunomodulators [6–9].

Lipids comprise a wide range of molecules, grouped in several classes and subclasses, with vast differences in size, shape, and molecular weight [10]. This broad heterogeneity is reflected in their varied functionality such as energy reservoirs (triglycerides), as structural components of the cell membrane (phospholipids, cholesterol, glycolipids, etc.) [11] or as intra- and intercellular signalling molecules (lipoproteins, sphingolipids, ceramides, etc.) [7, 12–14]. Lipids also play a key role in the regulation and development of several immune mechanisms. In this regard, the implication of lipid metabolism in diseases that involve systemic and chronic inflammation such as diabetes, atherosclerosis, and cancer, has been thoroughly investigated [9, 15, 16]. The biosynthesis of several haematopoietic and immune cells such as platelets, red blood cells, monocytes, and neutrophils, generally relies on lipids as modulators of stem cell differentiation and as an energy source [17]. Alternative M2 macrophage activation and differentiation is regulated by fatty acid oxidation to fuel OXPHOS and induce anti-inflammatory progression [18–20]. Focusing on adaptive immunity, B cell proliferation and survival require the use of mitochondrial OXPHOS and TCA cycle metabolic pathways [21, 22], while fatty acids and cholesterol synthesis allow activation-induced differentiation, proliferation, and growth of T cells by increasing the expression of the sterol regulatory element binding protein 1 and 2 [23–26]. Additionally, IL7 induces the synthesis of triacylglycerides to drive catabolic functions of memory CD8 + T cells, enhancing their self-renewal mechanisms and survival [27–31]. Moreover, Sphingosine-1-phosphate mediates leukocyte activation and migration through Ca^2+^ release and induces cytoskeleton reorganization to maintain vascular integrity [32–35]. Interaction between fatty acids and immunity also depends on fatty acid chain length: while short-chain fatty acids promote the formation of regulatory T cells, long-chain fatty acids promote the differentiation of Th1 and Th17 cells [23, 36].

Besides its crucial role in animal immunity and health, lipid metabolism is also a major determinant of production performance, carcass characteristics, and product quality in pig production. Previous studies discovered that the known breed-specific patterns of fat deposition are driven by differences in metabolic mechanisms of lipid absorption, oxidation, and biosynthesis [37–39]. These mechanisms have an impact in production performance by modulating lipid accumulation and energy availability. Triglycerides, phospholipids, and sterols are the main components of inter- and intramuscular fat [40, 41]. When considering the organoleptic and nutritional quality of the meat, the fatty acid profile of lipid deposits must be taken into consideration. It is well known that intramuscular fat content and distribution impacts the tenderness of pork products [42, 43]. Some fatty acids, such as oleic acid, have the capacity to impact the flavour of the meat [44].

Deepening the understanding of the complexity of tissues, cells, and molecular mechanisms requires system-level approaches, as addressed in [45] by integrating genomics, transcriptomics, proteomics, and metabolomics data. Following this trend, lipid studies have joined the ‘omics’ discipline, aiming to elucidate their molecular and functional diversity and create a full lipid atlas [46–48]. In the last decade, genomics, transcriptomics, and metabolomics have revealed intricate interactions and novel biomarkers that can potentially be used to improve relevant traits related to efficiency and resilience [49–53].

In this study, we focused on the plasma lipidome of healthy pigs to evaluate its genetic determinism and its relationships with health, welfare indicators, and production phenotypes.

## Methods

### Ethics

All experimental protocols and procedures with pigs were approved by the Institut de Recerca i Tecnologia Agroalimentàries (IRTA) Ethical Committee in accordance with the Spanish Policy for Animal Protection RD53/2013, which meets the European Union Directive 2010/63/EU for the correct practices and protection of the animals used in experimentation.

### Animal material and previous phenotypic parameters

The study was performed with a population of 300 healthy piglets (147 males and 153 females) from a commercial Duroc pig line. Animals originated from 122 litters born from 22 boars and 120 sows. Male piglets were surgically castrated at seven days of life. Females were immunocastrated by a triple injection of Vacsincel (Zoetis, Spain) at 95, 125, and 155 days of age, after sample collection. Two to four animals were selected from each litter (balancing gender when possible) and raised until the end of transition (~ 75 days of age) in the same maternity farm, distributed across six contemporary batches (50 ± 3 animals per batch). After weaning, animals were fed an ad libitum cereal-base commercial diet. Blood samples for the lipidome analyses and other blood measurements were collected at 60 ± 8 days of age via the external jugular vein into vacutainer tubes with or without anti-coagulants (Sangüesa S.A., Spain). At the moment of sampling, animals did not present any sign of infection or pathology.

A complete list of the phenotypes analysed in the present study can be found in Additional file 1, Table S1. Multiple health-related haematological, stress, and immunity phenotypes were measured, as described in Ballester et al. [54, 55]. Blood samples for these measurements were also collected at 60 ± 8 days. The health phenotypes included: plasma concentrations of different immunoglobulins (IgA, IgM and IgG), measured by ELISA; the serum concentrations of C-reactive protein (CRP), measured with a commercial ELISA kit, and of Haptoglobin (HP), measured by colorimetric assay; the percentage of phagocytic cells among lymphocytes and monocytes, as well as their phagocytic capacity, obtained by flow cytometry fluorescence analysis; haematological red blood cells related traits such as haematocrit (HTC), erythrocytes count (ERY), haemoglobin concentration (HB), mean corpuscular volume (MCV), mean corpuscular haemoglobin (MCH), and the counts of the white blood cells: leucocytes (LEU), monocytes (MON), eosinophils (EOS), neutrophils (NEU), and lymphocytes (LYM), which were measured as part of a complete hemogram; and the relative abundance among peripheral blood mononuclear cells (PBMCs) of Natural killer (NK), B and T lymphocytes and of the different T cells subpopulations γδ, Memory, Naïve, Helper, and Cytotoxic T lymphocyte (CTL) cells, obtained by flow cytometry analysis. The level of cortisol in plasma (CORTplasm) was measured by targeted liquid chromatography tandem mass spectrometry [56]. Additionally, hair samples were collected from the dorsal area of the neck behind the ears to measure the cortisol concentration in hair (CORThair) by ELISA. A detailed description of the procedures and platforms used for measurement of these haematological, immunity, and stress phenotypes can be found in Ballester et al. [54, 55].

After the growing-fattening period, animals were slaughtered between 181 and 228 days of age. Nine traits related to carcass and meat characteristics were obtained after slaughter, as described by Jové-Juncà et al. [5], including cold carcass weight (CW), backfat (BFT) and ham fat (HFT) thickness measurements, loin depth (LD), lean meat percentage of the carcass (LM) and of the ham (HLM), loin (LLM),and shoulder (SLM); and the pH in muscle measured at 24 h postmortem (pH24).

### Untargeted lipidomic data and profiling

Non-targeted lipidomics analysis was performed using the Agilent UHPLC-MS/QTOF 6545 equipment. Lipids were extracted from the samples by mixing 10 μL of plasma with 25 μL of water:methanol (20/80, v/v). Then, samples were mixed for 2 min and 250 μL of methyl tert-butyl ether containing internal standards of isotopically labelled lipids at 2.5 ug/mL. After, samples were sonicated for 30 min at 4 °C, 25 μL of Milli-Q water was added and mixed for 2 more min. Samples were centrifuged at 3,000 rpm at 4 °C for 10 min and the supernatant was aliquoted into liquid chromatography vials.

Lipids were analysed using the method of [57], using 10 μL of the extracted sample and separated in a 1.8 μm particle 100 × 2.1 mm id Waters Acquity HSS T3 column (Waters, Mildord, MA) heated at 55 °C. Liquid chromatography flow was 0.4 ml/min using phase A (water/acetonitrile, 60/40, v/v, 10 mM ammonium acetate) and phase B (isopropanol/acetonitrile, 90/10, v/v, 10 mM ammonium acetate). The gradient started at 40% of mobile phase B and reached 100% B in 10 min and was then held for 2 min. The system was switched back to 60% of mobile phase B and equilibrated for 3 min. Duplicate runs of each sample were performed to acquire positive and negative electrospray ionized lipid species data in a TOF mode, operated in full-scan mode from 100 to 1700 m/z in an extended dynamic range (2 GHz), using N_2_ as nebulizer gas (5 L/min, 350 °C). The capillary voltage was set at 3,500 V, with a scan rate of 1 scan/s. Continuous infusion was used for in-run calibration of the mass spectrometer, using an extra spray with masses 121.050873, 922.009798 (positive ion mode) and 119.036320, 966.000725 (negative ion mode).

Data were acquired and quality assessment was performed using the MassHunter Data Analysis Acquisition and Qualitative Software (Agilent Technologies). Batch-processing feature extraction was run using DA reprocessor and Mass Profiler Professional in the Agilent MassHunter Qualitative Software. Selected features had a minimum of 2 ions and multiple charge states were permitted for lipid species. Features were aligned using a retention time window of 0.1% ± 0.25 min and a mass window of 30.0 ppm ± 2.0 mDa. Molecular features were selected only if present in 70% of quality controls, resulting in 1144 molecules. The signal corresponded to relative intensity values normalized by internal deuterated lipid standards of TG and PE for positive and negative ionization, respectively (Avanti Polar Lipids, Cat. #86092 and #860374) and was corrected for individual bias using a LOESS approach [58, 59]. Finally, lipidome data were filtered for retention time above 3 to reduce the presence of small non-lipid species, obtaining a final list of 982 lipid molecules with signal in all analysed individuals.

Each lipid metabolite was named according to its ion mode, mass-to-charge ratio (m/z), and retention time (RT), using the following nomenclature system: “L_(Ion Mode)_(Neutral Mass)_(RT)”. The complete list of lipidic features analysed is in Additional file 2, Table S2.

Features defined by exact mass and RT were searched against the HMDB [60] and LIPID MAPS databases (accuracy < 30 ppm) [61]. For a reduced group of features (120 out of 982), the obtained lipid identities were compared to retention time of the authentic labelled internal standards that were added, and identities were confirmed by MS/MS by comparing the MS/MS spectrums using manual spectral comparison and LipidMatch, an R-based tool for lipid identification [62].

### Exploratory analyses

Basic descriptive statistics of the lipidome abundances were computed. Systematic non-genetic putative effects on the lipidome were tested by linear models with sex and batch as fixed effects, and normality of residuals was checked using the Shapiro–Wilk test. Most lipidome abundances (and their residuals) did not follow a normal distribution and various data transformations for normalizing the lipidome data were tested. The rank-based inverse normal transformation yielded the best results by normalizing the greatest number of lipid abundances and this transformation was applied to all lipidome abundances using the RNOmni package. All subsequent analyses were performed on this normalized dataset.

Relationships among the lipidome abundances and between the lipidome and the immunity, health, and carcass phenotypes were first explored using correlation analysis. Pairwise phenotypic correlations (r_p_) between the residuals (after correcting for the fixed effects of sex and batch) of the normalized abundance of lipid metabolites and with the immunity, health, and carcass phenotypes were obtained using the R software [63]. Corrplot clustering of the R software and hierarchical cluster analysis of the PermutMatrix software [64] were used to visualize the correlation matrix.

### Estimation of heritabilities and genetic correlations

Heritabilities for the normalized abundances of lipid species, and their genetic correlations with health and production phenotypes were estimated using Bayesian analyses performed by Gibbs sampling under bivariate animal models. The program *gibbs2f90* of the BLUPF90 family software [65] was used for all analyses to obtain the marginal posterior distributions of the variance components and of the corresponding heritabilities and genetic correlations. The following two-trait animal model was used for any combination of a lipid metabolite abundance (trait 1) and a health- or production- related trait (trait 2):

1 $$\left[\begin{array}{c}{\mathrm{y}}_{\mathrm{t}1}\\ {\mathrm{y}}_{\mathrm{t}2}\end{array}\right]=\left[\begin{array}{cc}{\mathrm{X}}_{\mathrm{t}1}& 0\\ 0& {\mathrm{X}}_{\mathrm{t}2}\end{array}\right]\left[\begin{array}{c}{\upbeta }_{\mathrm{t}1}\\ {\upbeta }_{\mathrm{t}2}\end{array}\right]+\left[\begin{array}{cc}{\mathrm{Z}}_{\mathrm{t}1}& 0\\ 0& {\mathrm{Z}}_{\mathrm{t}2}\end{array}\right]\left[\begin{array}{c}{\mathrm{u}}_{\mathrm{t}1}\\ {\mathrm{u}}_{\mathrm{t}2}\end{array}\right]+\left[\begin{array}{c}{\mathrm{e}}_{\mathrm{t}1}\\ {\mathrm{e}}_{\mathrm{t}2}\end{array}\right]$$

where ${\mathbf{y}}_{\mathrm{t}1}$ and ${\mathbf{y}}_{\mathrm{t}2}$ are the vectors of phenotypic observations for trait 1 and trait 2, respectively; ${{\boldsymbol{\upbeta}}}_{\mathrm{t}1}$ and ${{\boldsymbol{\upbeta}}}_{\mathrm{t}2}$ are the vectors of systematic (fixed) effects on each trait, including sex (2 levels) and batch (6 levels), and ${\mathbf{X}}_{\mathrm{t}1}$ and ${\mathbf{X}}_{\mathrm{t}2}$ the correspondent incidence matrices; ${\mathbf{u}}_{\mathrm{t}1}$ and ${\mathbf{u}}_{\mathrm{t}2}$ are the vectors of animal genetic additive random effects on traits 1 and 2, and ${\mathbf{Z}}_{\mathrm{t}1}$ and ${\mathbf{Z}}_{\mathrm{t}2}$ the corresponding incidence matrices; and ${\mathbf{e}}_{\mathrm{t}1}$ and ${\mathbf{e}}_{\mathrm{t}2}$ are the vectors of residual errors for each trait. A random litter effect was not included in the model for convergence issues as data structure (one litter by dam) did not allow adequately separate genetic from litter effect.        

The (co)variance matrix of random genetic effects was defined as follows:

2 $$ {\mathrm{Var}}\left[ {\begin{array}{*{20}c} {{\mathbf{u}}_{{{\mathrm{t}}1}} } \\ {{\mathbf{u}}_{{{\mathrm{t}}2}} } \\ \end{array} } \right] = \left[ {\begin{array}{*{20}c} {{\mathbf{A}}\sigma_{{{\mathrm{u}}1}}^{2} } & {{\mathbf{A}}\sigma_{{{\mathrm{u}}1,{\mathrm{u}}2}} } \\ {{\mathbf{A}}\sigma_{{{\mathrm{u}}1,{\mathrm{u}}2}} } & {{\mathbf{A}}\sigma_{{{\mathrm{u}}2}}^{2} } \\ \end{array} } \right] = {\mathbf{A}} \otimes \left[ {\begin{array}{*{20}c} {\sigma_{{{\mathrm{u}}1}}^{2} } & {\sigma_{{{\mathrm{u}}1,{\mathrm{u}}2}} } \\ {\sigma_{{{\mathrm{u}}1,{\mathrm{u}}2}} } & {\sigma_{{{\mathrm{u}}2}}^{2} } \\ \end{array} } \right] $$

where ${\upsigma }_{\mathrm{u}1}^{2}$ and ${\upsigma }_{\mathrm{u}2}^{2}$ are the additive genetic variances of traits 1 and 2, respectively, ${\upsigma }_{\mathrm{u}1,\mathrm{u}2}$ is the genetic covariance between the traits, and** A** is the numerator relationship matrix on the basis of pedigree (1141 individuals, five generations). Random residual errors, assumed independent between individuals, were assumed to have the following (co)variance matrix:

3 $$ {\mathrm{Var}}\left[ {\begin{array}{*{20}c} {{\mathbf{e}}_{{{\mathrm{t}}1}} } \\ {{\mathbf{e}}_{{{\mathrm{t}}2}} } \\ \end{array} } \right] = \left[ {\begin{array}{*{20}c} {{\mathbf{I}}\sigma_{{{\mathrm{e}}1}}^{2} } & {{\mathbf{I}}\sigma_{{{\mathrm{e}}1,{\mathrm{e}}2}} } \\ {{\mathbf{I}}\sigma_{{{\mathrm{e}}1,{\mathrm{e}}2}} } & {{\mathbf{I}}\sigma_{{{\mathrm{e}}2}}^{2} } \\ \end{array} } \right] = {\mathbf{I}} \otimes \left[ {\begin{array}{*{20}c} {\sigma_{{{\mathrm{e}}1}}^{2} } & {\sigma_{{{\mathrm{e}}1,{\mathrm{e}}2}} } \\ {\sigma_{{{\mathrm{e}}1,{\mathrm{e}}2}} } & {\sigma_{{{\mathrm{e}}2}}^{2} } \\ \end{array} } \right] $$

where ${\upsigma }_{\mathrm{e}1}^{2}$ and ${\upsigma }_{\mathrm{e}2}^{2}$ are the residual variances of traits 1 and 2, respectively, ${\upsigma }_{\mathrm{e}1,\mathrm{e}2}$ is the residual covariance between the traits, and **I** is an identity matrix.

Chains of 100,000 samples were run for each analysis, with a burn‐in of 10,000 rounds and sampling every 10 iterations to reduce autocorrelations. At any sample, heritabilities for the two traits and the genetic correlation between them were derived from the sampled variance components as: ${\widehat{\mathrm{h}}}_{\mathrm{t}1}^{2}= {\widehat{\upsigma }}_{\mathrm{u}1}^{2}/({\widehat{\upsigma }}_{\mathrm{u}1}^{2}+{\widehat{\upsigma }}_{\mathrm{e}1}^{2})$, ${\widehat{\mathrm{h}}}_{\mathrm{t}2}^{2}= {\widehat{\upsigma }}_{\mathrm{u}2}^{2}/({\widehat{\upsigma }}_{\mathrm{u}2}^{2}+{\widehat{\upsigma }}_{\mathrm{e}2}^{2})$ and ${\widehat{\mathrm{r}}}_{\mathrm{g}}= {\widehat{\upsigma }}_{\mathrm{u}1,\mathrm{u}2}/\left({\widehat{\upsigma }}_{\mathrm{u}1}{\widehat{\upsigma }}_{\mathrm{u}2}\right)$. Posterior means and standard deviations (SD) of the posterior distributions of the genetic parameters (heritability and genetic correlations with other phenotypes) were obtained for all measures of lipid species. The highest posterior density region at 95% not including zero was the criterion to consider the heritability estimate as highly probable. The posterior probability of the genetic correlation being > 0.2 (for positive estimates) or < -0.2 (for negative estimates) was computed and correlations were considered significantly different from zero when this probability was > 0.70.

## Results

### Descriptive statistics of the porcine plasma lipidome

We obtained the measured quantities for a total of 982 lipidic metabolites characterized by their specific mass-to-charge (m/z) ratio and retention time (RT). Summary statistics of the analysed plasma lipidome data is presented in Table 1 and descriptive statistics for the abundance of each individual lipid metabolite are in Additional file 2, Table S2. Positive ionization mode detected a total of 696 lipid structures, with m/z spectrum ranging from 66.0474 to 2334.632. The remaining 286 lipid structures were found in the negative ionization mode, with m/z spectrum ranging from 246.1979 to 1505.279. Prior to normal transformation of the data, mean abundances of the identified lipid metabolites ranged from 0.44 (for metabolite L_P_543.4503_7.399974) to 2.2 (for metabolite L_N_625.4971_7.50472), and the coefficient of variation (CV) of each lipid abundance within the population ranged from 5 (for metabolite L_P_747.7199_7.715224) to 1100% (for L_N_406.2289_3.653818). Subsequent analyses were performed on data normalized by a rank-based inverse normal transformation, which involved standardization of the different lipidome abundances.

**Table 1:** Statistics* of filtered porcine plasma lipidome: neutral mass, retention time, and abundance metrics

	All lipid features	Positive Ion Mode	Negative Ion Mode
	N = 982	N = 696	N = 286
Neutral Mass	720.92 (66.05 – 2334.63)	703.61 (66.05—2334.63)	763.05 (246.2—1505.28)
Retention Time	8.56 (3.05 – 11.32)	8.78 (3.05—11.32)	8.03 (3.07—11.29)
Mean lipid abundance	0.915 (0.443 – 2.198)	0.908 (0.443 – 1.513)	0.933 (0.58—2.198)
SD of lipid abundance **	0.474 (0.049 – 12.244)	0.472 (0.049 – 5.269)	0.477 (0.059—12.244)
CV (%) ***	52.7 (5.0 – 1118.3)	53.3 (5.0—682.2)	51.3 (5.9—1118.3)

^*^Mean (minimum – maximum) values. **SD: Standard deviation. ***CV: Coefficient of variation

Putative lipid mass identification of these molecules revealed a wide range of lipid classes that belong to the main lipid groups fatty acyls, glycerolipids, glycerophospholipids, ceramides, and steroids. Table 2 shows the summary of lipid classes that were putatively assigned to the lipidome features by mass similarity; the detail of the lipid class(es) assigned to each metabolite is in Additional file 2, Table S2. Mass similarity identification under the set mass window of ± 30.0 ppm (or the mass tolerance range of ± 0.01) reported up to seven different putative lipid classes for a given lipidic feature. Only 477 lipid features were assigned to a single lipid class, while 140 molecules lacked a reported class and were set as unknown. The most abundant putative lipid classes assigned among all lipidic features were triacylglycerols (15% of assignments), glycerophosphocholines (9%), diacylglycerols (8%), glycerophosphoethanolamines (8%), and fatty acids/esters (8%). When focusing on lipid features with a single putative identification, the triacylglycerol class remained as the abundant lipid class (33% of lipids features that were assigned to a unique lipid class), followed by diacylglycerols (12%). The class of 103 lipid molecules could be confirmed by MS/MS comparison (Table 2). Most of these belonged to the most abundant lipid groups glycerolipids (especially triacylglycerols) and glycerophospholipids (especially glycerophosphocholines), but several sphingolipids (ceramides), fatty acyls (fatty acids), and sterols, and one prenol lipid were also validated.

**Table 2:** Putative identification of lipid classes in the porcine plasma lipidome by mass similarity

Lipid Class	Lipid Group	All lipid features N = 982	Lipids with unique class N = 477	Lipids identified by MS/MS* N = 103
Tri(acyl|alkyl)glycerols	Glycerolipids	236	159	30
Glycerophosphocholines	Glycerophospholipids	135	31	21
Glycerophosphoethanolamines	Glycerophospholipids	127	27	6
Di(acyl|alkyl)glycerols	Glycerolipids	122	56	11
Fatty acids/esters	Fatty Acyls	119	25	4
Glycerophosphates	Glycerophospholipids	84	13	1
Glycerophosphoserines	Glycerophospholipids	74	15	
Ceramides	Sphingolipids	66	39	8
Hexosyl ceramides	Sphingolipids	64	10	5
Wax esters	Fatty Acyls	57		
Glycerophosphoglycerols	Glycerophospholipids	56	13	1
Sphingomyelins	Sphingolipids	49	8	4
Sterols, inc. bile acids	Sterols	44	13	4
Cholesterol esters	Sterols	34	2	
Short fatty esters	Fatty Acyls	33		
PE-ceramides	Sphingolipids	25	4	2
Mono(acyl|alkyl)glycerols	Glycerolipids	25	5	
Glycerophosphoinositols	Glycerophospholipids	22	10	1
N-acyl ethanolamines	Fatty Acyls	19	5	
FA estolides	Fatty Acyls	18		
Sphingoid bases	Sphingolipids	16	1	
Dihexosyl ceramides	Sphingolipids	14		
PI-ceramides	Sphingolipids	14	1	
Acyl carnitines	Fatty Acyls	12	3	
Lyso-Glycerophosphocholines	Glycerophospholipids	12	3	2
Lyso-Glycerophosphoethanolamines	Glycerophospholipids	12		
Sulfatides	Sphingolipids	12	5	2
Other lipid classes marginally represented (< 10 features) from different lipid groups: Fatty Acyls, Sphingolipids, Glycerolipids, Glycerophospholipids and Prenol lipids		10 to 1	9 to 1	1 prenol lipid

^*^Lipids whose class was confirmed by MS/MS comparison

The matrix of estimated phenotypic correlations between the abundance of the different lipid molecules of the plasma lipidome revealed important associations between them. Correlations coefficients ranged from -0.75 to 0.99, with 307 out of the 982 metabolites being highly correlated (over 0.8) with one or more other lipids. When clustering the correlations [see Additional file 3, Table S3], metabolites with similar m/z tended to group together, i.e. they were positively correlated with each other.

### Lipidome associations with health and production traits

Estimates of phenotypic correlations between the abundance of different lipid species and a plethora of immunity, stress, and carcass quality parameters are provided in Additional file 4, Table S4. A total of 908 metabolites were significantly correlated at phenotypic level with at least one of the considered traits. Despite significant, these phenotypic correlation estimates were generally low to medium. Only 292 metabolites showed correlations greater than 0.20 (in absolute value) with one or more immunity or production traits. The strongest positive and negative phenotypic correlation estimates were found for the serum concentration of HP with the abundance of metabolites L_P_575.5644_8.642532 (r_p_ = 0.41) and L_N_713.5351_8.003355 (r_p_ = -0.43), respectively. Mass- based identification classified this first lipid as a ceramide or a wax ester, while the second molecule’s mass matched two different lipid classes, a Hexosyl ceramide and a Glycerophosphoethanolamine.

Erythrocytes count was the trait that showed significant phenotypic correlations with the largest number (268) of lipid metabolites. Next, HP, the plasma concentration of IgM, the serum concentration of CRP, the percentage of γδ T lymphocytes, the MCV, and the LYM count also showed significant phenotypic correlations with a relevant number (> 200) of molecules in the lipidomic profile. The remaining white blood cell counts (LEU, EOS, NEU, and MON) showed significant phenotypic correlations with a moderate number of lipid metabolites (> 70). All white blood cells counts (LYM, LEU, EOS, NEU, and MON) had similar phenotypic correlations with several lipid molecules, highlighting the metabolites L_P_731.5468_7.411574 and L_P_896.687_9.786056, which had, respectively, strong positive and negative phenotypic correlations with white blood cell counts. Based on a lipid mass search, L_P_896.687_9.786056 was putatively annotated as a triacylglycerol, while lipid L_P_731.5468_7.411574 was confirmed by MS/MS comparison to be a phospholipid (PC(32:1)).

**Fig. 1:**
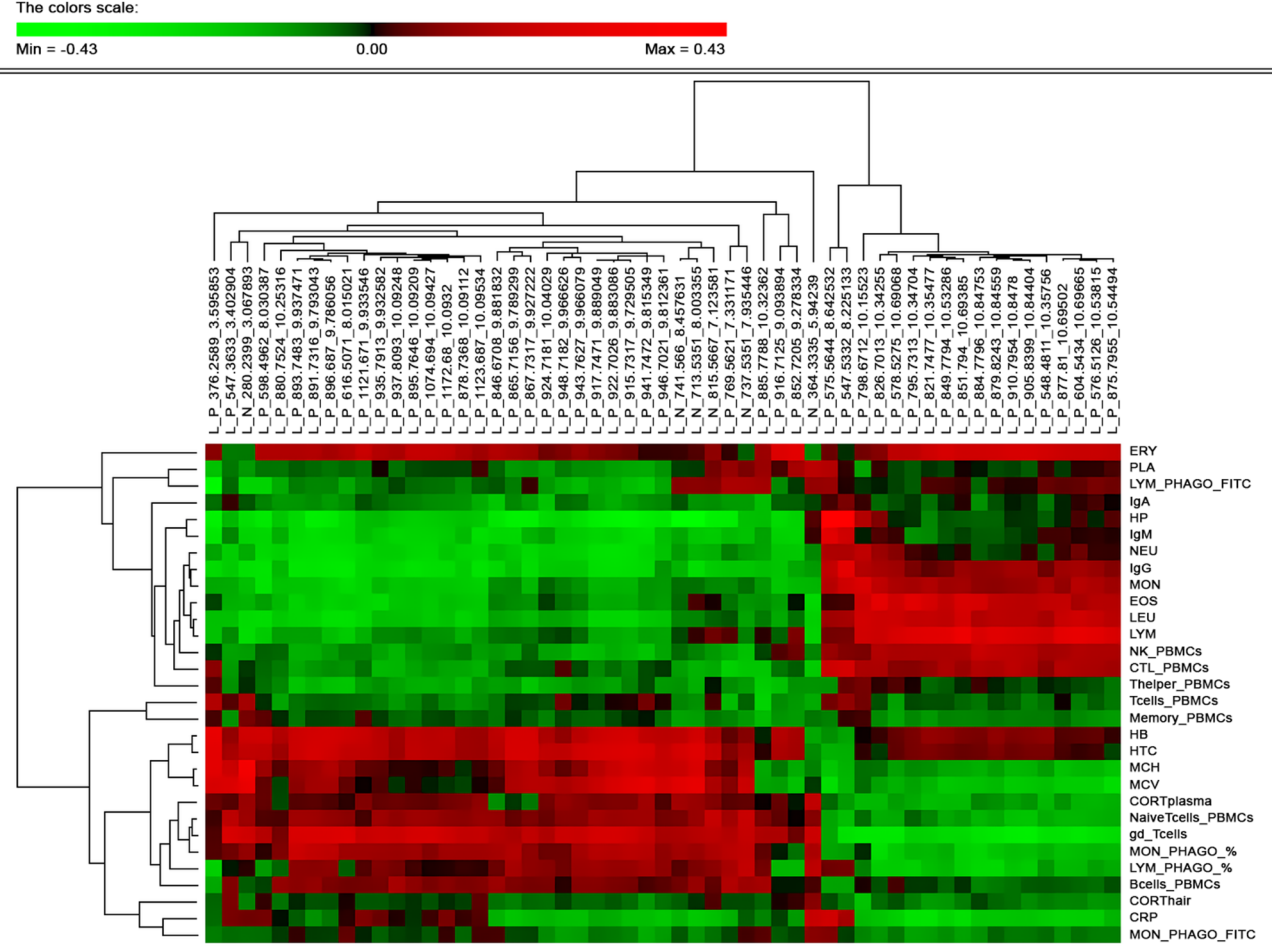
Estimates of phenotypic correlations between the plasma lipidome and immunity traits. Heatmap of phenotypic correlation coefficients between the health traits and the 55 lipid metabolites of lipidome with greatest number of significant phenotypic associations between them (> 11 for lipidic compounds). Lipid metabolites and immunity traits are grouped by hierarchical clustering. Abbreviations: Ig = Immunoglobulin; HP = haptoglobin; CRP = C-reactive protein; CTL = Cytotoxic T cell; NK = Natural killer cell; PBMC = Peripheral Blood Mononuclear Cells; gd_Tcells = gamma delta T cells; ERY = Erythrocytes; PLA = Platelets; LEU = Leucocytes; MON = Monocytes; LYM = Lymphocytes; EOS = Eosinophiles; NEU = Neutrophiles; HB = Haemoglobin; HTC = Haematocrit; MCH = Mean Corpuscular Haemoglobin; MCV = Mean Corpuscular Volume; CORT = Cortisol; FITC = Fluorescein isothiocyanate

A total of 55 lipid molecules that showed significant phenotypic correlations with 12 or more immunity traits were chosen for further analysis. Figure 1 shows the hierarchical cluster analysis of phenotypic correlations of these molecules with the health traits, revealing existence of two clusters of health traits. One cluster included the plasma concentrations of immunoglobulins (IgA, IgM and IgG), the serum concentration of HP, all white blood cells (LEU, EOS, NEU, MON and LYM) plus erythrocytes and platelet counts, the phagocytic capacity of lymphocytes, and the relative abundance among PBMCs of NK and several T cells subpopulations ( CTL and T helper cells). The second cluster included the serum concentration of CRP, the remaining haematological parameters related to red blood cells (MCV, MCH, HTC and HB), the percentage of phagocytic cells among both monocytes and lymphocytes and phagocytic capacity of monocytes, the relative amount of B cells, of T cells, and of several T cells populations (naïve, memory, and γδ T lymphocytes), and the levels of cortisol in both plasma and hair. A large number of lipid molecules showed opposite phenotypic correlations with these two clusters of health phenotypes, i.e. lipids that had positive correlations with the first cluster of health traits had negative correlations with the second cluster, and vice versa. When considering the putative classes of these lipids, we observed the main presence of phospholipids and glycerolipids.

**Fig. 2:**
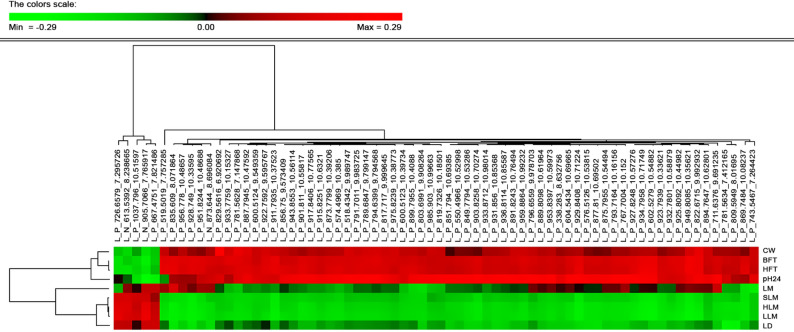
Estimates of phenotypic correlations between the plasma lipidome and production traits. Heatmap of phenotypic correlation coefficients for the 70 lipid metabolites significantly associated to at least seven carcass and meat traits. Lipid metabolites and production traits are grouped by hierarchical clustering. Abbreviations: carcass weight (CW), lean meat percentage (LM), ham lean meat percentage (HLM), loin lean meat percentage (LLM), shoulder lean meat percentage (SLM), backfat thickness (BFT), loin depth (LD), ham fat thickness (HFT) and pH 24 h semimembranosus (pH24)

Among the carcass and meat phenotypes measured at slaughter, pH at 24 h *postmortem* had significant phenotypic correlations with the largest number of molecules in the lipidome (249). A total of 70 metabolites revealed significant correlations with at least seven out of the nine carcass and meat trait phenotypes (Fig. 2). Cluster analysis of these correlations identified two groups of traits with opposed correlation patterns, with lean meat and loin depth being inversely correlated with the lipidome compared to the fat deposition, CW, and pH phenotypes. In general, the cluster of lipids that was positively correlated with fatness and negatively with lean meat was mainly composed of diacyl- (DGs) and triacyl-glycerides (TGs), while lipids that were positively correlated with lean meat and negatively with fatness had a higher number of potential ceramides and phospholipids.

### Heritability of lipid molecule abundance

**Fig. 3:**
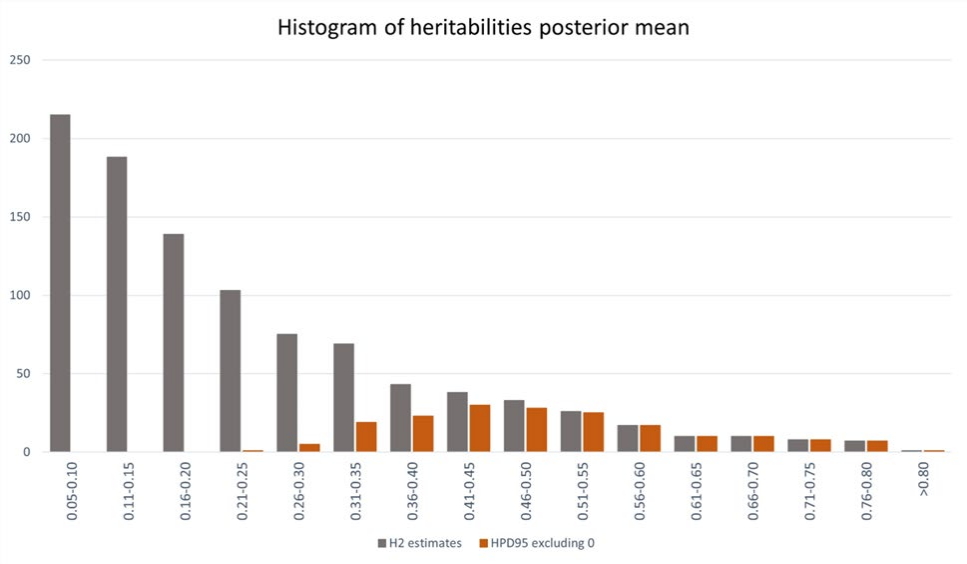
Distribution of the estimates of heritability of the plasma lipidome. Histogram of the posterior mean estimates of heritability for the abundance of 982 lipid molecules characterized in the porcine plasma lipidome; in orange those heritabilities whose highest posterior distribution at 95% (HPD95) did not encompass zero in any analysis

The genetic determinism of lipid metabolites was evaluated based on heritability estimates obtained from bivariate animal models. Figure 3 presents the histogram of mean heritability estimates for the 982 lipids, while the range of posterior means of the heritability of each metabolite in the different analyses is shown in Additional file 5, Table S5. The majority of lipids compounds had low to medium heritability estimates, but a total of 184 out of 982 compounds reported an HPD95% for heritability that did not encompass zero in any analysis. These most probable heritability estimates took values above 0.25. The most heritable metabolite was L_P_684.5827_10.89376, with posterior mean heritability ranging between 0.80 and 0.88. Considering the putative classification of the lipid features into lipid classes based on mass similarity, we found no evidence that any particular lipid class was consistently more or less heritable than other classes. Although the highest heritability estimates were mostly observed for TGs, DGs, phospholipids, ceramides, and some cholesterol species, these same classes also included molecules with some of the lowest heritability estimates.

Estimates of heritability for the immunity and production traits from the bivariate analyses with the lipidome are summarized in Additional file 5, Table S5. In general, these estimates were similar to those obtained with univariate models by Ballester et al*.* [54, 55] and Jové-Juncà et al*.* [5] with medium to high heritability estimates for most traits. Among the immune traits, IgA and IgG in plasma, the percentage of phagocytic lymphocytes and their phagocytic capacity, EOS count, and the relative abundance of T lymphocytes among PBMCs presented particularly high heritability estimates (> 0.7). In the case of production phenotypes, heritability estimates around 0.7 were obtained for all carcass traits related with growth and fatness.

### Genetic correlations of the lipidome with immunity and production traits

The estimated genetic correlations (r_g_) between the lipidome and phenotypes related to health and production performance are shown in Additional file 6, Table S6. Overall, the posterior means of the genetic correlations were much higher than the corresponding phenotypic correlations estimates, with absolute values ranging from 0 to 0.94, but the genetic correlation estimates also showed high posterior SD. Because of this, a conservative threshold was set to declare a genetic correlation as probable: having a posterior density of being either greater than 0.2 (positive correlations) or less than -0.2 (negative correlations) greater than 70, 80, or 90%. A total of 925 lipid molecules met this requirement with one or more health and production traits, for a total of 7,592 significant genetic correlations, and will be presented hereafter (Table 3).

**Table 3:** Number of correlated metabolites for each health and production trait

PHENOTYPES	Estimated genetic correlation ^a^			PHENOTYPES	Estimated genetic correlation ^a^		
	*p* > 0.7	*p* > 0.8	*p* > 0.9		*p* > 0.7	*p* > 0.8	*p* > 0.9
IgA	114	17	0	γδ T-lymphocytes subpopulation	247	116	7
IgM	205	73	9	B lymphocytes	67	4	0
IgG	129	16	2	T lymphocytes	188	48	3
HP	131	32	7	Memory T cells	155	30	0
CRP	116	18	2	Naïve T cells	218	33	2
Leukocytes count	75	13	1	T Helper cells	117	30	3
Lymphocytes count	119	35	1	Natural killer cells	96	24	1
Monocytes count	91	0	0	CTL cells	41	5	0
Eosinophils count	115	24	0	Cortisol in plasma	276	97	12
Neutrophils count	172	22	0	Cortisol in hair	358	129	15
Haematocrit	158	41	2	Cold carcass weight	189	60	5
Erythrocytes count	194	75	18	Lean meat	345	137	25
Haemoglobin	200	64	8	Ham lean meat	339	129	21
MCV	175	40	3	Loin lean meat	342	144	20
MCH	171	44	2	Shoulder lean meat	310	107	12
Platelets count	282	109	9	Loin depth	249	68	4
Phagocytic Lymphocytes	234	89	7	Backfat thickness	310	125	7
Phagocytic Monocytes	104	11	0	Ham-fat thickness	289	102	4
Phagocytic capacity of Lymphocytes	224	73	6	pH 24 h semimembranous	229	68	2
Phagocytic capacity of Monocytes	218	67	0				

^a^Estimated genetic correlation; *p* > 0.7, *p* > 0.8 and *p* > 0.9 refers to the probability above 70%, 80% and 90% of being either higher than 0.2 (for positive correlations) or lower than -0.2 (for negative correlations)

Among the health phenotypes, those related to the percentage of phagocytic lymphocytes and their phagocytic capacity were particularly associated with the lipidome, showing more than 200 relevant genetic correlations. Among them, the strong genetic correlations (r_g_ ≥ 0.8) of the lipids L_P_717.5319_7.775038 and L_P_481.3172_3.928623 with the phagocytic traits of both lymphocytes and monocytes stood out. A lipid mass search identified several phospholipids within a 0.01 margin of the molecule L_P_717.5319_7.775038, while for L_P_481.3172_3.928623, a fatty acyl carnitine and several lysophospholipid species were found to match. Other health phenotypes showing a large number of highly probable genetic correlations with the lipidome were the proportion of γδ T lymphocytes, serum HP concentration, erythrocyte and platelet counts, and MCV. Conversely, leukocyte-related traits showed less significant genetic correlations with the lipidome, with the exception for the relative abundance of some T cells subpopulations (memory, helper, and naïve T cells).

Regarding stress-related phenotypes, cortisol concentration in plasma also showed significant genetic correlations with a large number of lipid molecules. In fact, the posterior mean for the genetic correlation of CORTplasm with the lipid L_N_748.5516_9.479212 (r_g_ = -0.94) was found to be the overall strongest genetic correlation of any phenotype with the lipidome. Mass search identified this lipid as a potential glycerophosphoglycerol. Meanwhile, L_N_795.5157_5.967228 had the strongest positive genetic correlation (r_g_ = 0.87) between the lipidome and cortisol levels in plasma. For this metabolite, we obtained two potential lipid identifications, either as a glycerophosphoserine or a sulfatide. Significant genetic correlations with a large number of lipid molecules were also obtained for cortisol concentration in hair, in general matching the directionality of those estimated for CORTplasm. However, 12 lipids showed opposed genetic correlations with these two cortisol measurements: seven showed positive genetic correlations with cortisol in hair and negative correlations with cortisol in plasma, while the remaining five showed the inverse pattern. A wide variety of lipid classes were identified as potential identities for these 12 lipids, with glycerolipids, phospholipids, and ceramides being the most repeated putative annotations. Among them, L_P_362.3031_3.055466 and L_P_593.5049_8.216685 had the strongest negative genetic correlation with cortisol in plasma and the strongest positive genetic correlation with cortisol in hair. These molecules were putatively identified as a *N*-acylethanolamine and MS/MS validated as a diacyl-glyceride, respectively. Also relevant was the lipid L_P_930.242_3.687853, which had a positive genetic correlation with cortisol in plasma and a negative correlation with cortisol in hair and was putatively annotated as an acyl-CoA.

**Fig. 4:**
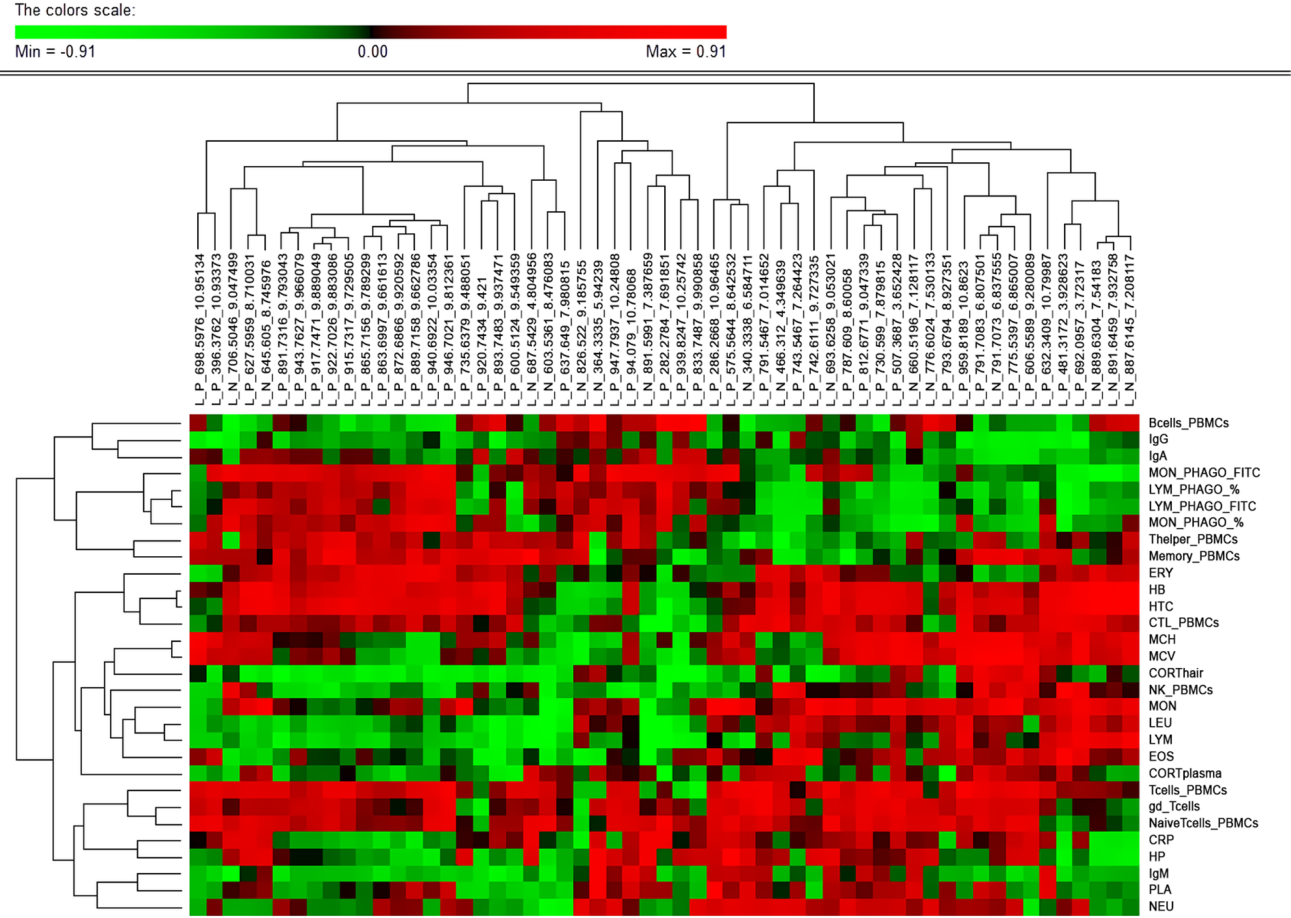
Genetic correlation estimates between the plasma lipidome and immunity phenotypes. Heatmap of genetic correlation estimates between health traits and the 55 lipid metabolites with greatest (> 10) number of significant genetic associations between them. Lipid metabolites and immunity traits are grouped by hierarchical clustering. Abbreviations: Ig = Immunoglobulin; HP = haptoglobin; CRP = C-reactive protein; CTL = Cytotoxic T cell; NK = Natural killer cell; PBMC = peripheral blood mononuclear cells; gd_Tcells = gamma delta T cells; ERY = Erythrocytes; PLA = Platelets; LEU = Leucocytes; MON = Monocytes; LYM = Lymphocytes; EOS = Eosinophiles; NEU = Neutrophiles; HB = Haemoglobin; HTC = Haematocrit; MCH = Mean Corpuscular Haemoglobin; MCV = Mean Corpuscular Volume; CORT = Cortisol; FITC = Fluorescein isothiocyanate

Figure 4 shows the hierarchical cluster of genetic correlation estimates with health traits for lipid molecules that showed significant genetic correlations with more than ten health phenotypes. Different from the clustering based on phenotypic correlations, the clustering of health traits based on genetic correlations with the lipidome did not offer well-differentiated clusters of traits. However, small clusters of traits were evident that had similar genetic correlations with part of these lipids. These included, among others, the cluster formed by the two acute phase proteins concentrations (HP and CRP), the cluster of red blood cell related traits (HTC, HB and ERY), the cluster of all phagocytosis related traits, and the cluster formed by white cell counts (LEU, EOS, MON and LYM).

**Fig. 5:**
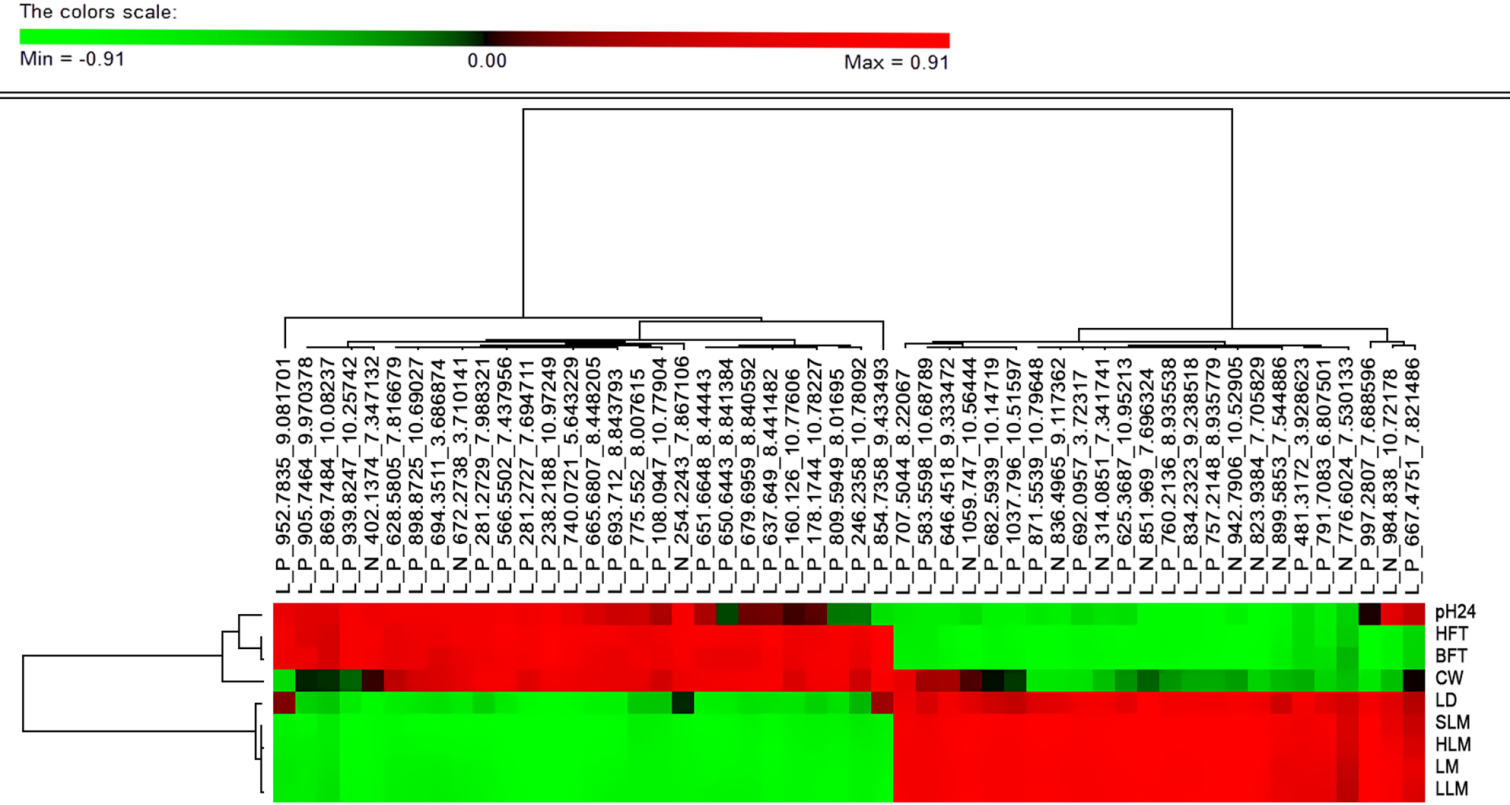
Genetic correlation estimates between the plasma lipidome and production phenotypes. Heatmap of genetic correlation estimates for the 52 lipid metabolites significantly associated to at least eight carcass and meat traits. Lipid metabolites and production traits are grouped by hierarchical clustering. Abbreviations: carcass weight (CW), lean meat percentage (LM), ham lean meat percentage (HLM), loin lean meat percentage (LLM), shoulder lean meat percentage (SLM), backfat thickness (BFT), loin depth (LD), ham fat thickness (HFT) and pH 24 h semimembranosus (pH24)

With regard to carcass and meat quality traits, 659 lipidic molecules had a significant genetic correlation with at least one of these production-related traits (Additional file 6, Table S6). Both fatness and lean meat measures showed relevant genetic correlations with more than 250 lipid molecules, observing an opposite pattern of correlations between these two groups of traits, i.e., lipids that correlating positively with fat deposition correlated negatively with lean meat, and vice versa. Figure 5 shows the hierarchical cluster of genetic correlations of the nine carcass traits with metabolites that had seven or more significant genetic correlations and with an opposite pattern of genetic correlations of lipidome with fatness versus lean content. Among these, the lipid L_P_238.2188_10.97249 had the strongest negative genetic correlations with lean meat traits and the strongest positive genetic correlations with both fat measurements. Mass search and MS/MS validation identified this molecule as the lipid 9-decenoylcholine. Conversely, the lipid L_P_997.2807_7.688596, which was putatively identified as a fatty acyl CoA, showed the strongest negative genetic correlation with BFT and had high positive genetic correlations with the lean meat content measures. For the other carcass traits, a total of 189 metabolites had significant genetic correlations with CW, and 229 metabolites had significant genetic correlations with pH24. In general, both CW and pH24 tended to follow the pattern of genetic correlations as observed for the fat depot phenotypes, while few metabolites matched the pattern of correlations observed for lean meat content. In that regard, we highlight the lipids L_P_707.5044_8.22067 and L_P_830.6341_10.05227, which had similar high positive genetic correlation estimates with CW and the four lean meat phenotypes, while having strong and negatively genetic correlations with the fat measures BFT and HFT. Mass search for these molecules revealed a high number of glycerolipids and glycerophospholipids with similar weights.

## Discussion

This is a pioneer study about porcine plasma lipidome that explores its genetic determinism and its association with health and production phenotypes at both the phenotypic and genetic level. A total of 982 lipidic compounds were detected by mass spectrometry in plasma samples of healthy young Duroc pigs. The metabolites obtained in the lipidome analysis revealed to have a wide range of m/z and RT, implying that highly diverse lipid species are composing the plasma of pigs. The most abundant lipid groups among the lipid features in the porcine plasma lipidome were glycerolipids (mainly triacylglycerols but also diacylglycerols), glycerophospholipids (mainly glycerophosphocholines and glycerophosphoethanolamines), and fatty acyls (mainly fatty acids); despite less abundant, sphingolipids, sterols, and one prenol lipid were also identified. The phenotypic correlations among the abundance of the detected metabolites revealed strong links between metabolites with similar m/z values, suggesting that these lipids may be structurally and/or functionally related.

A large number of the identified lipid molecules showed associations with several health-related traits, mostly with the concentration of haptoglobin in serum. Estimated phenotypic correlations were, however, low to moderate, in line with previous studies that showed that highly multifactorial and complex traits tend to have low phenotypic correlations between them [66, 67]. Clustering of lipids that were most associated with health traits revealed two groups of health phenotypes and two groups of lipid molecules that had opposite correlation patterns. As a group, plasma immunoglobulins, serum HP concentration, leukocytes, platelets, and erythrocytes counts, as well as NK, T-helper, and CTL cells, showed opposite correlations with the lipidome compared to the group composed by serum CRP concentration, haematological traits related to red blood cells, except for ERY, the percentage of phagocytic cells, B and T cells, naïve, memory, and γδ T lymphocytes, plus cortisol in plasma and hair. It is worth highlighting the opposite phenotypic correlations with lipidome that were observed for the acute phase proteins CRP and HP, and for the different lymphocyte populations (γδ T cells *vs* NK and CTL T cells). This pattern of associations could indicate diverse and opposite functions of some metabolites in immune regulation, as well as in cell composition, fate, and function [29, 31, 68]. The putative lipid classification revealed a great variability in the lipid classes that formed these two clusters, being mainly composed of phospholipids and glycerolipids. This variability implies that the observed phenotypic correlations with the health traits are specific to each lipid molecule and not class dependent. These results are consistent with previous findings highlighting that different lipid species within the same lipid class can be functionally diverse [69].

Among the health traits most associated with the lipidome, white blood cell counts correlated similarly with the two clusters of lipid molecules. The lipid molecules L_P_731.5468_7.411574 and L_P_896.687_9.786056 had, respectively, strong positive and negative phenotypic correlations with leukocyte phenotypes and were annotated as a validated phospholipid and a putative triacylglycerol, respectively. Phospholipids are abundant in the cellular membrane of various immune cells, influencing their activity by changing cell adherence, cell to cell communication, and cell metabolic activity [70, 71]. Moreover, phospholipid metabolism is key to a correct signalling-mediated regulation of the immune response [14, 23]. Triglycerides are mainly known for their role as cellular energetic reservoirs, yet they play a significant role in general immunity. The effects of triglycerides on inflammatory response and modulation of immunity cells are complex, involving both stimulatory and inhibitory mechanisms, depending on their fatty acid composition [68, 72]. In previous studies, medium-chain and saturated fatty acids showed to have pro-inflammatory properties [73], while triglycerides composed of long-chain polyunsaturated fatty acids, especially omega 3 fatty acids, proved to have an anti-inflammatory effect by suppressing several inflammation mechanisms [74, 75].

The genetic determinism of the porcine blood lipidome was analysed by estimating the heritability of the abundance of the lipid species detected in plasma. Heritability estimates showed wide variability across molecules, with posterior mean heritabilities distributed throughout the entire parametric space. While most compounds exhibited low-to-medium heritability estimates, almost 20% of heritability estimates took medium-to-high values and were detected as highly probable. These results support a certain genetic determinism of the concentration levels of lipids in plasma, beyond their dependence on dietary intake and other non-genetic factors. A similar range of heritability estimates was found in previous studies conducted in humans and mice [69, 76–82], which showed that genetic determinism of lipid species differs even within the same lipid class.

At the genetic level, estimates of correlations between lipidome and health and performance traits were generally larger than corresponding correlations at the phenotypic level. The estimates of genetic correlations obtained in this study should, however, be interpreted with caution. On the one hand, the limited sample size resulted in large posterior SD of these estimates (i.e. low precision). On the other hand, we cannot discard the presence of a litter environmental effect on some of these phenotypes, which could lead to overestimation of the genetic component if not fitted. Unfortunately, the available data did not allow us solving more parameterized models that included random litter effects. In any case, this study reports a first picture about the genetic correlations between the lipidome and a plethora of health, stress, and productive phenotypes that is worthy to be analysed.

Among the evaluated health traits, those related to the proportion of phagocytic cells and their phagocytic capacity were particularly associated with several lipid molecules at the genetic level. Phagocytosis plays a crucial part in the elimination of foreign substances and pathogens. A fast and efficient phagocytic response depends on lipid-mediated recognition mechanisms [83]. Furthermore, lipids contribute to cytoskeleton remodelling by providing the structural support and dynamic flexibility that is required for the phagosome formation. For instance, lipid L_P_717.5319_7.775038 was estimated to have high positive genetic correlations with all phenotypes related to phagocytosis. Putative identification of this lipid revealed several glycerophospholipids. Phospholipids are highly bioactive lipids that play a role in the regulation of phagocytic activity through various mechanisms [84]. Depending on the structure and fatty acid composition, the phospholipids located in the cell membrane of phagocytic cells act as signalling molecules, regulate the release of cytokines, and are essential for cytoskeleton remodelling, phagosome formation, and engulfment efficiency [85–87]. In turn, the lipid L_P_481.3172_3.928623 was estimated to be highly and negatively correlated at the genetic level with the percentage of phagocytic lymphocytes and monocytes, and with their activity level. A fatty acyl carnitine and several lysophospholipids species have a similar weight as this metabolite. As observed for other lipid classes, the lysophospholipid group included very diverse lipids with various functions with respect to inflammation and immune response [88, 89]. In fact, Khandelwal et al. [90] demonstrated the importance of the fatty acid chain length of lysophosphatidylserines for the activation and modulation of the phagocytic activity of macrophages. Fatty acyl carnitines are required for the transport of fatty acids into the mitochondria during the cycle of β-oxidation [91, 92]. The function of M2 macrophages depends on fatty acid oxidation [18, 92]. Thus, a higher percentage of these phagocytic cells would increase the demand for lipid mitochondrial oxidation and a decrease in the demand for fatty acyl carnitine. Moreover, the accumulation of this metabolite in the cellular environment can shift the metabolic pathway choice and indirectly alter the synthesis of other lipids, such as sterols and phospholipids, thereby altering phagocytic activity [93].

Phenotypes related to animal stress, such as cortisol levels in both plasma and hair, also had highly probable genetic correlation estimates with the lipidome, despite their low phenotypic correlations. Several lipid molecules showed similar genetic correlation estimates with cortisol concentrations in both plasma and hair, suggesting their potential as biomarkers of the animal’s general stress levels. However, a group of metabolites showed opposing genetic correlations with the levels of cortisol in hair (indicator of chronic stress) versus in plasma (indicator of acute stress), including phospholipids, glycerolipids and ceramides. Metabolic pathways, including lipid metabolism, shift in response to stress through various mechanisms that could vary depending on whether the stress is acute or chronic [94, 95] and these metabolic changes could affect some lipid molecules differently. On the one hand, acute stress activates the hypothalamic–pituitary–adrenal axis and the rapid release of cortisol, which triggers adipocyte lipolysis [96]. Additionally, acute stress leads to increased production of specific lipidic metabolites, such as eicosanoids and other free fatty acids [97]. On the other hand, during chronic stress, carbohydrates become the predominant source of energy, leading to lipogenesis and an increase in the levels of several lipidic metabolites, such as glycerolipids and phospholipids [98, 99]. In the same way that stress modifies the cellular metabolism of the organism, the metabolic profile may have the capacity to modulate the susceptibility and response of animals to stress. Growing evidence suggest that sphingolipid metabolism has a core role in stress response [100, 101]. Reginato et al*.* [101] reported that ceramide supplementation, which results in higher circulating levels of this lipid, was accompanied with hypothalamus dysregulation, cellular inflammation, endoplasmic reticulum stress, and lipotoxicity. Previous studies have also shown that supplementation with phospholipids, such as phosphatidylethanolamines and phosphatidylcholines, has an hormetic anti-oxidative effect to the stress-induced generation of reactive oxygen species (ROS), thereby improving the genetic response to stress and increasing survival [102, 103]. Another group of anti-oxidative lipid compounds are acylethanolamines, which have been shown to reduce the production of cytokines, NO, and ROS, and to have antidepressant-like results in mild chronic stressed mice [104, 105]. In this regard, the annotation of the metabolite L_P_362.3031_3.055466 as a N-acylethanolamine NAE(20:5) is noteworthy, among the lipids with positive genetic correlations with cortisol in hair (chronic stress) and negative genetic correlations with cortisol in plasma,. Considering the anti-inflammatory role of these lipidic molecules in oxidative stress, we could hypothesise that a higher general presence of this lipid may have a protective effect against chronic stress. Lastly, among the lipids that had positive genetic correlations with cortisol in plasma (acute stress) but negative correlations with cortisol in hair, the molecule L_P_930.242_3.687853 was annotated as an acyl-CoA, a lipid class that regulates metabolic enzymes and is involved in lipid metabolism [106–108]. It can be hypothesised that this lipid molecule plays a role in the shift of metabolic pathway to lipid oxidation to provide the required energy during acute stress.

Relevant correlations were also observed of circulating lipid compounds at a young age with final carcass and meat traits, at both the phenotypic and genetic levels, despite the time gap between lipidome characterization (60 days of age) and carcass phenotype measurements (slaughter age). In general, the carcass traits correlated with the same lipidic compounds but showing opposite patterns when comparing the correlations with lean meat versus those with fat depot phenotypes, in concordance with the divergence between these two groups of traits [5, 109, 110]. These findings suggests that the circulating lipidomic profile at a young age could contribute to inferring the animal’s subsequent production performance in terms of lean growth and capacity of accumulating and distributing fat deposits during the fattening period.

Among lipids with the strongest genetic correlations with carcass and meat traits, two molecules stood out among the rest: L_P_238.2188_10.97249, which was highly positively correlated with fat deposition phenotypes, and L_P_997.2807_7.688596, which was strongly positively associated with lean meat growth. The lipid L_P_238.2188_10.97249 was identified to be the fatty acyl 9-decenoylcholine, whose genetic correlation with fat depots in the final stages of production could suggest that this lipid plays some role in fat distribution and accumulation during fattening period. The L_P_997.2807_7.688596 lean meat associated metabolite was classified as an acyl-CoA, a group of lipid compounds involved in the degradation and storage of free long chain fatty acids. Considering the observed pattern of genetic correlations of this lipid with lean meat and fat deposit phenotypes, we can infer that this specific Acyl-CoA may play a role in the metabolism of lipids, specifically in the b-oxidation of free fatty acids, reducing the accumulation of lipids in the body [43, 108]. It is also worth mentioning the lipid metabolites L_P_707.5044_8.22067 and L_P_830.6341_10.05227, which both had positive genetic correlations with both lean content and carcass weight. These results suggest that these metabolites are potential indicators of lean growth rate in pigs. Lastly, several lipid molecules were found to be genetically correlated with pH at 24 h postmortem. Most of these lipids showed similar genetic correlation patterns with pH24 as with carcass weight and fatness traits, as could be expected from the previously reported positive genetic correlations between these traits [5]. The pH reduction rate depends on the energetic status of the muscle at slaughter and its subsequent postmortem metabolism [111, 112]. For instance, availability of a major concentration of glycogen for rapid degradation generates higher concentrations of lactate and H + , accelerating the pH decline and resulting in paler, soft, and exudative meat [113]. A plausible hypothesis behind genetic correlations with lipidome is that certain lipid molecules and profiles, even at early production stages, may influence biochemical mechanisms involved in postmortem pH decline, thus serving as potential indicators of the physicochemical and organoleptic characteristics of the resulting meat.

## Conclusions

This research represents the first study on porcine blood lipidome composition and its phenotypic and genetic associations with health and production traits. The study demonstrates the genetic determinism of the porcine blood lipidome and provides an initial picture of the phenotypic and genetic correlations of the lipidome and a plethora of health, stress, and carcass and meat traits in swine. Furthermore, the study identifies potential lipid biomarkers that are associated with these traits, offering valuable insights for future research and applications in animal health and productivity. However, further analyses using larger and multibreed populations are necessary to enhance the power and representativity of these results.

## Supplementary Information

Below is the link to the electronic supplementary material.

Supplementary Material 1

Supplementary Material 2

Supplementary Material 3

Supplementary Material 4

Supplementary Material 5

Supplementary Material 6

## Data Availability

The original contributions presented in the study are included in the article/Supplementary Material. Further inquiries can be directed to the corresponding authors upon reasonable request.
